# Mid-Trimester Cervical Length Screening in Routine Practice: Short-Cervix Prevalence, Preterm Birth and the Shift of the Progesterone Counselling Threshold from 20 to 25 mm in a 15-Year Single-Centre Cohort of 20,168 Singleton Pregnancies

**DOI:** 10.3390/jcm15218310

**Published:** 2026-10-25

**Authors:** Maria Cezara Mureșan, Marius Bogdan Mureșan, Dan Dumitrașcu Biriș, Daniela-Eugenia Popescu

**Affiliations:** 1 Department of Obstetrics and Gynecology, Faculty of Medicine, “Victor Babeș” University of Medicine and Pharmacy, 300041 Timișoara, Romania; 2 Maternal-Fetal Medicine Center, 300331 Timișoara, Romania; 3 Department of Obstetrics-Gynecology and Neonatology, “Victor Babeș” University of Medicine and Pharmacy, 300041 Timișoara, Romania

**Keywords:** cervical length, preterm birth, second-trimester ultrasound, vaginal progesterone, short cervix, universal screening, neonatal outcome, clinical practice

## Abstract

**Background/Objectives:** Vaginal progesterone reduces preterm birth in women with a short mid-trimester cervix, and the treatment threshold has moved from 15–20 mm to 25 mm; how this change reached everyday practice is rarely documented. We describe cervical length (CL) distribution, short-cervix prevalence, preterm-birth and neonatal outcomes and the evolution of progesterone counselling in routine screening. **Methods:** Retrospective cohort of singleton pregnancies with a CL measurement at 16^+0^–24^+6^ weeks in one Romanian fetal-medicine centre (2011–2026). CL was measured transabdominally and, when <30 mm, transvaginally (FMF criteria). Documented counselling was extracted from the structured report text by keyword search; delivery outcome was available for 20.3% of pregnancies, so outcome analyses describe the outcome-ascertained subgroup. **Results:** Among 20,168 pregnancies, median CL was 35.8 mm; CL was <25 mm in 1.92% (95% CI 1.74–2.12) and <15 mm in 0.60%, and five times more frequent after a previous late miscarriage or preterm birth. In 4087 pregnancies with known delivery, birth < 34 weeks rose from 1.9% at CL ≥ 35 mm to 26.1% at <15 mm; CL < 25 mm carried an adjusted odds ratio of 9.1 (4.6–17.9) for birth < 34 weeks and birth weight < 1500 g in 8/92 (8.7%) vs. 41/3894 (1.1%), yet identified only 12% of births < 34 weeks (AUC 0.59); 88% (95% CI 79–93%) of births < 34 weeks followed a CL ≥ 25 mm. An explicit progesterone mention at 20–24.9 mm rose from 49.2% in 2011–2016 to 73.3% in 2017–2026 (*p* = 0.002); a data-driven change-point search located the shift in 2017–2019 (81% of clustered bootstrap samples), as a level change without trend, and the period effect persisted after adjustment for population composition (OR 5.0, 2.3–10.9). Before 2017 the operational threshold was ≈22 mm. **Conclusions:** A short cervix is rare but carries nine-fold adjusted odds of early preterm birth; screening identifies a small very-high-risk group rather than most preterm births. The structured report captured, in real time, the migration of documented counselling to the full ≤25 mm range between 2017 and 2019; neonatal findings are exploratory.

## 1. Introduction

Preterm birth remains the leading cause of neonatal death and of long-term childhood morbidity, affecting an estimated 9.9% of live births worldwide in 2020 and about 8% of births in Europe [1,2]. Among the many candidate predictors, sonographic cervical length (CL) in the mid-trimester is the most extensively validated: in the landmark NICHD Maternal–Fetal Medicine Units study, the risk of spontaneous preterm birth increased continuously as CL decreased, with a relative risk of about 6 for a CL at or below the 10th percentile (25 mm) [3]. Large Fetal Medicine Foundation (FMF) screening series subsequently showed that a CL ≤ 15 mm at 22–24 weeks is found in about 1.5% of routinely screened women and precedes most deliveries before 32 weeks [4,5,6].

The clinical relevance of CL screening depends on the availability of an effective intervention. Vaginal progesterone reduced early preterm birth in women with a CL ≤ 15 mm in the FMF trial [7] and in women with a CL of 10–20 mm in the PREGNANT trial [8]. An individual-patient-data (IPD) meta-analysis published in 2012 had already suggested a benefit across the whole ≤ 25 mm range [9], and the evidence was consolidated by the updated IPD meta-analysis of five trials published online in November 2017 and in print in 2018, which showed a 38% reduction in birth < 33 weeks and a parallel reduction in neonatal morbidity [10]; the conclusion was subsequently confirmed by the EPPPIC IPD collaboration and by network meta-analysis [11,12]. The Society for Maternal-Fetal Medicine, FIGO and ISUOG now consider universal transvaginal CL screening at 18–24 weeks reasonable, with vaginal progesterone offered for a CL ≤ 25 mm [13,14,15], whereas earlier practice, shaped by the inclusion criteria of the original trials, had often used lower thresholds of 15–20 mm.

Whether universal screening actually lowers population preterm birth rates is less certain: single-centre pre/post studies reported modest reductions [16,17,18] or none [19], recent meta-analyses conclude that the effect is small and of low certainty [20,21], and the largest individual trial of progesterone in high-risk women was negative [22]; nevertheless, modelling studies suggest that universal screening followed by progesterone is cost-saving [23]. Part of the uncertainty stems from the low sensitivity of a single mid-trimester CL for all preterm births in low-risk women, illustrated by the nuMoM2b cohort, in which a CL ≤ 25 mm identified only 8% of spontaneous preterm births in nulliparous women [24]. Another practical issue is the route of measurement: transabdominal assessment is more acceptable and faster but overestimates CL and misses a substantial proportion of short cervices, which is why a two-step strategy—transabdominal screening followed by transvaginal measurement when the transabdominal value falls below a safety margin—has been proposed [25,26,27,28].

Data from Central and Eastern Europe are scarce. The only multicentre Romanian report focused on the treatment of women who already had a short cervix [29]; no Romanian study has described the distribution of CL, the prevalence of a short cervix and the resulting preterm birth and neonatal outcomes in a routine-screening population. Moreover, although the move from a 15–20 mm to a 25 mm treatment threshold is one of the clearest examples of evidence consolidation and guideline change in obstetrics over the past decade, we are not aware of any study that has documented, from contemporaneous clinical records, how and when this change was adopted at the bedside.

We therefore analysed 15 years of routine mid-trimester CL screening in a single fetal-medicine centre with three aims: (i) to describe the distribution of CL and the prevalence of a short cervix in a largely unselected Romanian routine-screening population screened with a two-step transabdominal–transvaginal protocol; (ii) to quantify the association of CL with preterm birth and neonatal outcome and the screening performance of the usual thresholds; and (iii) to use the structured text of the ultrasound report to document the evolution of counselling for vaginal progesterone, cerclage and pessary, and in particular the shift of the progesterone threshold from 20 to 25 mm.

## 2. Materials and Methods

### 2.1. Study Design and Setting

This was a retrospective cohort study of prospectively recorded routine clinical data from the Maternal-Fetal Medicine Center, Timișoara, a private fetal-medicine unit in western Romania that performs first- and second-trimester screening for a largely unselected routine-screening population of self-referred and physician-referred women; the unit also receives a minority of referrals for suspected fetal or cervical pathology. All examinations since January 2009 are recorded in a structured obstetric ultrasound database (Astraia version 30.1.2, Astraia Software GmbH, Munich, Germany) that stores biometry, cervical measurements, the FMF preterm-birth risk calculation, the free-text report sent to the referring physician, and, when available, pregnancy outcome. The study is reported according to the STROBE recommendations for cohort studies.

### 2.2. Cervical Length Protocol

Throughout the study period the centre applied a two-step protocol to every woman attending the mid-trimester anomaly scan. CL was first measured transabdominally with a partially filled bladder as part of the routine examination. Whenever the transabdominal measurement was <30 mm, or whenever the cervix could not be visualised adequately—irrespective of the apparent transabdominal value—a transvaginal measurement was performed with an empty bladder in the sagittal plane, without fundal pressure, taking the shortest of three measurements from the internal to the external os, in accordance with FMF and ISUOG criteria [15,30,31]; the transvaginal value was then recorded as the CL of the examination. Consequently, in this cohort all values < 30 mm are transvaginal measurements confirmed under standardised conditions, whereas most values ≥ 30 mm are transabdominal. The presence of funnelling was recorded when assessed. The FMF algorithm combining CL and obstetric history [6] was used to compute patient-specific risks of delivery before 28, 31, 34 and 37 weeks, which are stored in the database. Examinations were performed on Voluson 730 Expert, E6, E8, E10, S10 and Expert 22 systems (GE Healthcare, Zipf, Austria) by fetal-medicine specialists holding FMF certificates of competence.

### 2.3. Counselling Policy and Its Documentation

Women with a short cervix were counselled at the time of the scan and received a written report addressed to the referring physician. This report is generated from a structured template in which the measured CL, the associated risk and the recommended management are stated in standardised wording (“the cervix measures … mm, which represents an increased risk of preterm birth …; the following is indicated: vaginal micronised natural progesterone 200 mg daily until 34 weeks; surgical treatment—cervical cerclage; cervical pessary; re-assessment of cervical length”). The centre’s policy, based on the trials available at the time, was to recommend vaginal progesterone for a CL below approximately 20 mm in the early years and, as the evidence for the whole ≤25 mm range was consolidated [10] and incorporated into guidelines [13,14,15], for any CL ≤ 25 mm; cerclage and pessary were discussed as options for very short cervices or progressive shortening, in line with the evidence available for these interventions [32,33]. The exact year of the change was not recorded administratively and is one of the objects of this study.

The recommendations were extracted from the report text by keyword search performed on the pseudonymised database. For the revised analysis the search was repeated on the three free-text fields of the examination record (the report letter, the diagnosis field and the recommendation field) after normalisation of case, diacritics and non-standard characters, using the pattern progest/progestr/utrogest/arefam/crinone/lutinus/prolut* for vaginal progesterone, cerclaj/serclaj/cerclage for cerclage and pesar* for pessary. Compared with the search used in the originally submitted version (two fields, no character normalisation), this identified 11 additional progesterone mentions among 20,168 index examinations (9 at CL < 25 mm); all counts in this version are from the repeated search and the small resulting differences are listed in the response to the reviewers. The primary indicator, “explicit progesterone mention”, encompasses initiation, continuation, conditional use or discussion as an option. From 2023 the wording of the report template changed in two ways: a generic phrase (“initiation of treatment according to the prescription [Rp]”) replaced the named drug in part of the reports, and a narrative of the FMF risk calculation (“low/increased risk of preterm birth”) was added. Because the prescription itself is not stored in the database, we defined a secondary, broader indicator (“documented management”: explicit progesterone mention, or the generic prescription phrase in the context of cervical re-assessment advice, or cerclage, or pessary) and report it as a sensitivity analysis; it adds 15 pregnancies with CL < 25 mm, almost all from 2024 to 2025, and it should be read as documentation of some cervical management rather than as a verified progesterone prescription. Extracted counselling reflects the documented recommendation and not adherence, dispensing or the actual performance of a procedure. In particular, a keyword indicating cerclage or pessary was interpreted only as documentation that the procedure had been discussed as a management option in the counselling letter; it does not indicate that it was recommended as first-line treatment or performed. The report letter was available for 20,114 of the 20,168 index examinations (385 of the 388 with CL < 25 mm); the absence of a drug mention therefore reflects the content of an available report rather than a missing report.

### 2.4. Cohort Definition

The database covers examinations from 1 January 2009; because CL measurement entered the routine protocol during 2011, the study period is 2011 to 31 August 2026 and all cohort figures refer to this period, whereas the flow diagram (Figure S1) starts from the full database. Examinations belonging to the same woman were grouped into pregnancies using the patient identifier and the examination dates and gestational ages (a new pregnancy was assumed when the interval between examinations exceeded 210 days or gestational age decreased by more than 3 weeks). The index examination was the first CL measurement at 16^+0^–24^+6^ weeks of each pregnancy; the minimum CL recorded in this window was also retained. Multiple pregnancies (recorded chorionicity or more than one fetus) were excluded. Implausible CL values (≤0 or >70 mm) were treated as missing.

### 2.5. Outcomes

Pregnancy outcome is entered into the database from the delivery ward of the affiliated hospital, from the referring physician or from the patient. Delivery outcome was defined as a recorded live birth, intrauterine death or neonatal death with a known gestational age at delivery ≥ 22 weeks; terminations and miscarriages were not counted as deliveries. Birth weight, Apgar score and mode of delivery are reported with the number of deliveries in which each item was recorded as the denominator; PPROM is reported over all recorded deliveries. Preterm birth was defined as delivery before 37, 34, 32 and 28 completed weeks, irrespective of onset, because the onset of labour was documented in fewer than 10% of deliveries. Because CL predicts spontaneous rather than medically indicated preterm birth, a sensitivity analysis approximated spontaneous preterm birth from the available surrogates (“surrogate-defined probable spontaneous preterm birth”): a preterm delivery was classified as probably spontaneous when the mode of delivery was vaginal, vacuum or emergency caesarean section, or when PPROM or spontaneous onset of labour was recorded, and as “probably indicated” when it was an elective caesarean section without any of these features; deliveries with unknown mode were retained in the all-cause endpoint only. This surrogate is not a validated spontaneous endpoint—an emergency caesarean section may be performed for pre-eclampsia, placental abruption or fetal compromise without spontaneous onset of labour—and a more restrictive definition limited to deliveries with documented spontaneous onset of labour or PPROM was therefore also examined. Neonatal variables (birth weight, 1-min Apgar score, neonatal-unit transfer) were recorded in a minority of deliveries, are reported with their own denominators and are considered exploratory. Birth weight, 1-min Apgar score, neonatal-unit transfer, preterm prelabour rupture of membranes (PPROM) and mode of delivery were analysed when recorded. Because outcome ascertainment was incomplete, the characteristics of pregnancies with and without a recorded delivery were compared, and all outcome analyses were restricted to pregnancies with a recorded delivery.

### 2.6. Statistical Analysis

Continuous variables are summarised as median (interquartile range, IQR) or mean ± SD and categorical variables as counts and percentages with exact (Clopper–Pearson) 95% confidence intervals (CIs). CL was categorised as <15, 15–19.9, 20–24.9, 25–29.9, 30–34.9 and ≥35 mm. For the descriptive comparison of counselling by CL category, the study period was divided into 2011–2016 and 2017–2026; we acknowledge that this cut-point was chosen with knowledge of the evidence timeline and of the annual data, and it is therefore treated as a descriptive simplification rather than a pre-specified hypothesis. To locate the change formally, we (i) profiled, in the 20–24.9 mm band, a single-step binomial model over all candidate change years 2013–2024 and selected the year maximising the log-likelihood; (ii) assessed the stability of the selected year by resampling women (with all their pregnancies) 2000 times with replacement (seed 20261017) and recording the distribution of the selected year; (iii) fitted an interrupted-time-series logistic model with linear pre-period trend, level change and slope change at the selected year; (iv) repeated the simple step comparison with 2016, 2018 and 2019 as alternative cut-points; and (v) adjusted the step effect for population composition (maternal age, nulliparity, previous spontaneous delivery at 16–36 weeks, CL and gestational age at the index scan, with and without linear calendar year). Bootstrap selection frequencies describe the stability of the data-driven change year and are not confidence intervals for a protocol date, and confidence intervals of the step models condition on the selected year. All analyses were repeated with the broader “documented management” indicator. The annual counselling rate at 20–24.9 mm is shown with exact 95% CI and a centred three-year moving average. Proportions of key interest, including the proportion of preterm births occurring at CL ≥ 25 mm, are given with exact 95% CI. The association between CL and preterm birth was assessed with the Spearman trend test across categories, with sensitivity, specificity, predictive values, likelihood ratios and relative risks for the 15, 20, 25 and 30 mm thresholds, and with the area under the receiver-operating-characteristic curve (AUC) with bootstrap 95% CI (1000 resamples). Logistic regression estimated the odds ratio (OR) of preterm birth per 5 mm decrease in CL and for CL < 25 mm, adjusted for maternal age, body-mass index, smoking, in vitro fertilisation, nulliparity, previous spontaneous delivery at 16–36 weeks (late miscarriage or preterm birth) and calendar year of the index scan as a continuous covariate (the originally submitted models used the binary period instead; both specifications are given in Table S4); nulliparity and previous delivery at 16–36 weeks were derived from the recorded obstetric history, and pregnancies with missing covariates were excluded from the models (complete-case analysis). The discrimination of the stored FMF risk for spontaneous birth < 34 weeks against our all-cause endpoint is reported in the Supplementary Materials (Table S8) for completeness only; because the target outcomes differ, it is not an evaluation of the algorithm and is not used in the main analysis. Analyses were performed in Python 3.11 (pandas 2.2, statsmodels 0.14, scikit-learn 1.5); two-sided *p* < 0.05 was considered significant.

### 2.7. Ethics

The study was conducted in accordance with the Declaration of Helsinki and approved by the Ethics Committee of the Maternal-Fetal Medicine Center, Timișoara, Romania (approval No. 1/2026 of 1 August 2026; framework approval No. 2/2026 of 7 September 2026; Addendum No. 1 to the framework approval, 8 September 2026, covering the present study); the three documents are successive extensions of a single authorisation for the retrospective secondary analysis of pseudonymised clinical data. Patient consent was waived because the study was a retrospective, non-interventional analysis of pseudonymised routine clinical data collected over more than 15 years, and only aggregate results are reported. Free-text reports were processed only in the pseudonymised environment and were not retained in the analytical dataset.

## 3. Results

### 3.1. Study Population

Of 57,009 examinations recorded in the database up to 31 August 2026, 35,463 included a valid CL measurement. After reconstruction of pregnancies, 20,636 pregnancies had at least one CL measurement at 16–24 weeks; 468 multiple pregnancies were excluded, leaving 20,168 singleton pregnancies in 17,966 women examined between 2011 and August 2026 (Figure S1). The index examination was performed at a median of 21.6 weeks (IQR 20.7–22.3), and 19,366 (96.0%) were at 18–24 weeks. Maternal characteristics are summarised in Table 1: median age was 30.6 years, median body-mass index 22.5 kg/m^2^, 63.3% of those with a recorded history were nulliparous, 3.6% had conceived by in vitro fertilisation, 16.3% smoked and 471 of 17,817 with a recorded history (2.6%) had a previous spontaneous delivery between 16 and 36 weeks (late miscarriage or preterm birth, the history category used by the FMF preterm-birth algorithm [6]).

### 3.2. Distribution of Cervical Length and Prevalence of a Short Cervix

**Figure 1:**
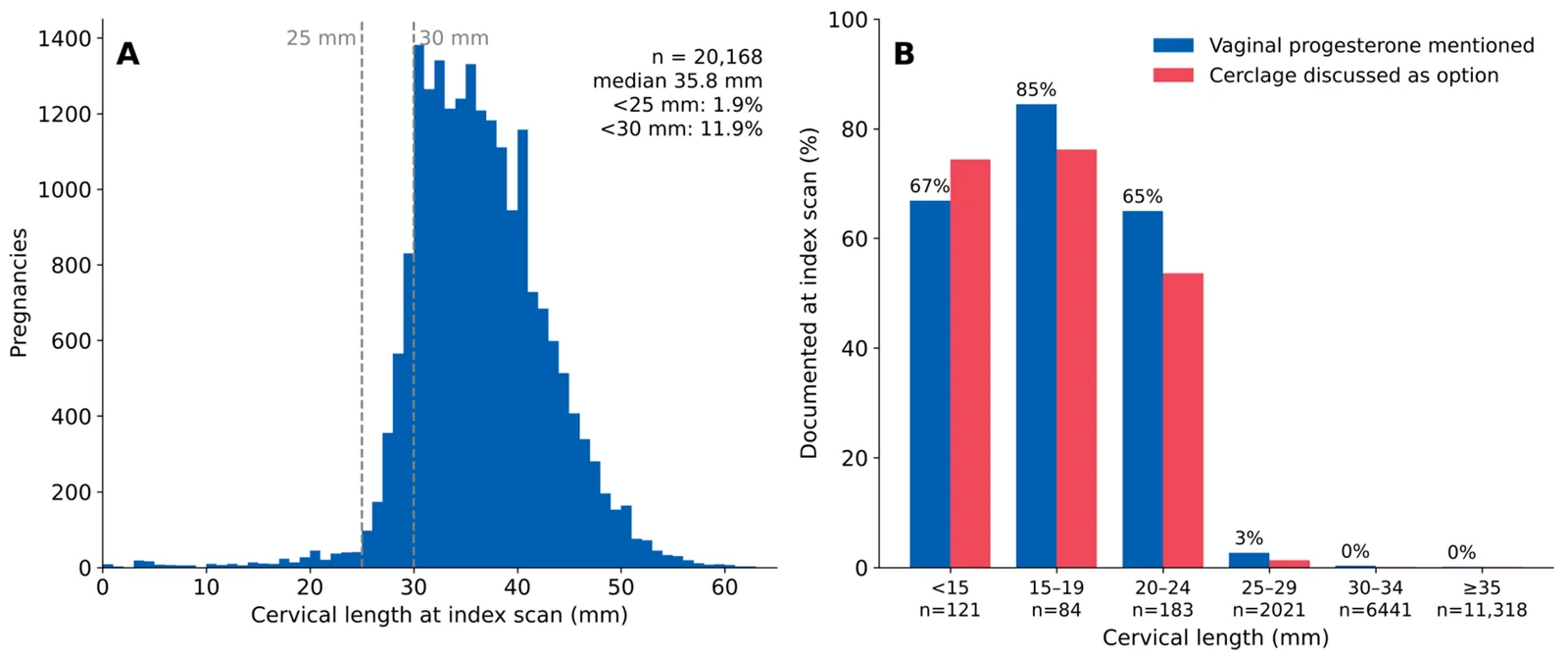
(**A**) Distribution of cervical length at the index scan in 20,168 singleton pregnancies; dashed lines mark 25 mm (short cervix) and 30 mm (trigger for transvaginal measurement). (**B**) Documented counselling at the index scan according to cervical length category: explicit mention of vaginal progesterone, and cerclage discussed as a management option (documentation of discussion only, not of recommendation or performance).

The distribution of CL at the index scan is shown in Figure 1A. Mean CL was 36.2 ± 6.5 mm; the 1st, 5th, 10th, 50th and 95th percentiles were 19.7, 27.9, 29.3, 35.8 and 47.0 mm. The distribution shows a discontinuity at 30 mm, the threshold of the two-step protocol, with a relative paucity of values between 25 and 29.9 mm (2021 pregnancies, 10.0%) compared with the 30–34.9 mm band (6441, 31.9%): values below 30 mm represent transvaginal re-measurement, which is on average shorter than the transabdominal estimate. Median CL was stable across the 16–24-week window (35.0–36.3 mm per completed week; 5th percentile 27–29 mm), so that no gestational-age adjustment was applied.

A CL < 25 mm was found in 388 pregnancies (1.92%, 95% CI 1.74–2.12), <20 mm in 205 (1.02%, 0.88–1.16), <15 mm in 121 (0.60%, 0.50–0.72) and <30 mm in 2409 (11.9%, 11.5–12.4). Using the minimum CL recorded at 16–24 weeks instead of the index value, 415 pregnancies (2.1%) had a CL < 25 mm. The annual prevalence of a CL < 25 mm was 3.2–4.4% in 2011–2013, when the newly available measurement attracted referred high-risk women, and 0.8–2.6% thereafter (Table S1). Funnelling was recorded as present in 76 of the 7219 pregnancies in which it was assessed (1.1%): in 55% of those with CL < 15 mm, 33% at 15–19.9 mm, 13% at 20–24.9 mm, 0.6% at 25–29.9 mm and ≤ 0.2% above 30 mm.

Obstetric history, recorded in the categories of the FMF algorithm (previous spontaneous delivery at 16–23 weeks, i.e., late miscarriage; at 24–33 weeks; at 34–36 weeks; after 36 weeks; nulliparous), was strongly associated with a short cervix (Table 2): a CL < 25 mm was present in 9.6% of women with a previous late miscarriage at 16–23 weeks, 9.9% with a previous birth at 24–33 weeks and 9.2% with a previous birth at 34–36 weeks, compared with 1.4% after a previous term birth and 2.0% in nulliparous women.

### 3.3. Documented Counselling by Cervical Length and the Shift of the Progesterone Threshold

An explicit mention of vaginal progesterone was documented in 81/121 (66.9%, 95% CI 57.8–75.2) women with a CL < 15 mm, 71/84 (84.5%, 75.0–91.5) at 15–19.9 mm and 119/183 (65.0%, 57.6–71.9) at 20–24.9 mm—cumulatively 271/388 (69.8%, 65.0–74.4) below 25 mm—but in only 55/2021 (2.7%) at 25–29.9 mm and in ≤0.3% above 30 mm (Figure 1B; Table 3). With the broader “documented management” indicator, the proportions were 66.9%, 86.9% and 72.1% (286/388, 73.7%, below 25 mm). Cerclage was discussed as a management option in 74.4%, 76.2% and 53.6% of the three short-cervix categories, and a pessary in 1.6–3.3%; in the templated letter, these procedures are listed among the options explained to the woman, not as the recommended treatment. Of the 415 pregnancies with a minimum CL < 25 mm, 57 (13.7%) had at least one further CL measurement within the 16–24-week window.

**Figure 2:**
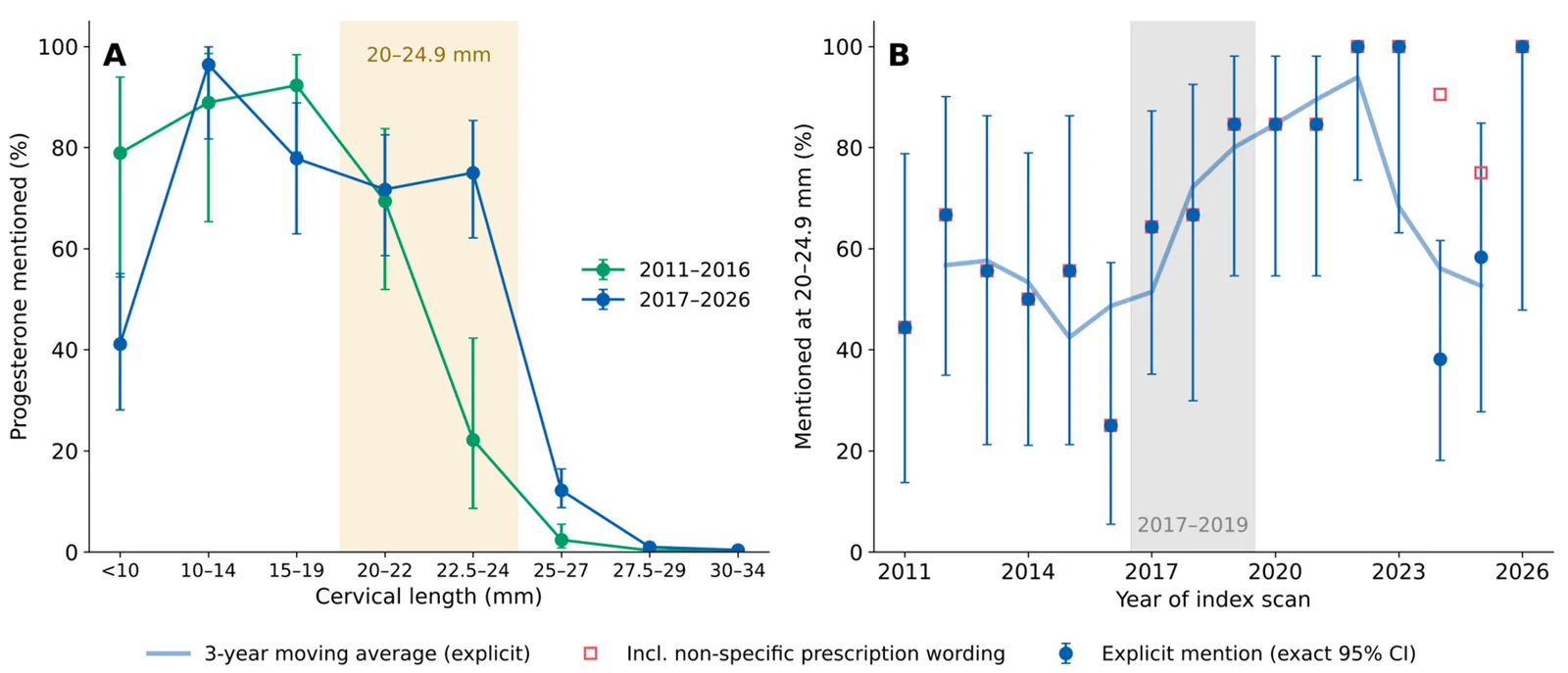
The shift of the progesterone counselling threshold. (**A**) Proportion of women with an explicit progesterone mention by 2.5–5 mm bands of cervical length in 2011–2016 and 2017–2026 (points, proportions; bars, exact 95% CI); the shaded band marks 20–24.9 mm. (**B**) Annual proportion of women with a cervical length of 20–24.9 mm with an explicit progesterone mention (points, exact 95% CI; 5–21 pregnancies per year, Table S3), with the broader documented-management indicator (open squares) and the centred three-year moving average of the explicit indicator; the grey band marks 2017–2019, the window in which the data-driven change-point search located the shift (Table S6). No policy-change year is assumed; 2026 includes January–August only.

The period comparison shows where the documented practice changed (Figure 2). Below 20 mm, a progesterone mention was already the rule in 2011–2016 (67/76, 88.2%) and did not increase afterwards (85/129, 65.9%). At 20–24.9 mm, in contrast, the proportion rose from 49.2% (31/63; 95% CI 36.4–62.1) in 2011–2016 to 73.3% (88/120; 64.5–81.0) in 2017–2026 (χ^2^ *p* = 0.002). Finer categorisation (Figure 2A) shows that before 2017 the operational threshold lay at about 22 mm—progesterone was mentioned for 69.4% of women with a CL of 20–22.4 mm but for only 22.2% of those with 22.5–24.9 mm—whereas from 2017 onwards the two sub-bands were counselled equally (71.7% and 75.0%). A modest extension into the 25–27.4 mm band also appeared (2.4% to 12.2%).

The annual series at 20–24.9 mm (Figure 2B; Table S3) is based on 5–21 pregnancies per year. The explicit mention was documented in 25.0% (3/12) in 2016, 64.3% (9/14) in 2017, 66.7% (6/9) in 2018, 84.6% (11/13) in each of 2019–2021 and 100% in 2022–2023; it fell to 38.1% (8/21) in 2024 and 58.3% (7/12) in 2025, when the generic prescription wording appeared in the reports, and with the broader indicator the 2024 and 2025 proportions were 90.5% (19/21) and 75.0% (9/12), so that the apparent late decline is a change of wording rather than of practice. The formal change-point search (Table S6) selected 2017 as the first year of the later period for the explicit indicator, with 2018 and 2019 practically indistinguishable (deviance differences 0.43 and 0.84), and 2019 for the broader indicator; in the clustered bootstrap, the selected year fell within 2017–2019 in 81.4% and 82.6% of samples, respectively. The interrupted-time-series model showed no pre-2017 trend (OR 0.82 per year, 0.61–1.11), a level increase at 2017 (OR 7.6, 1.9–30.6) and no change of slope thereafter (OR 1.13 per year, 0.81–1.57). The step effect was similar with 2018 or 2019 as cut-point (OR 2.7 and 2.6) and smaller with 2016 (OR 1.8, 0.94–3.54), and it persisted after adjustment for maternal age, nulliparity, previous spontaneous delivery at 16–36 weeks, CL and gestational age at the index scan (OR 5.0, 2.3–10.9; with linear calendar year added, 7.6, 1.9–30.5). The composition of the 20–24.9 mm band changed little between periods (median age 28.7 vs. 30.6 years; nulliparous 71% vs. 62%; previous preterm birth 15% vs. 7%; median CL 22.0 vs. 22.5 mm; Table S7). The lower rate of explicit progesterone mention at CL < 15 mm after 2017 (59.5%) reflects the composition of this group: two-thirds (56/84) had a CL < 10 mm, versus about half (19/37) before 2017, and in this extreme sub-band the letter named progesterone in only 41.1% (and cerclage in 57.1%), often documenting urgent referral for admission or cerclage instead of the templated recommendation, whereas at 10–14.9 mm progesterone was mentioned in 27/28.

### 3.4. Preterm Birth and Neonatal Outcome According to Cervical Length

Delivery outcome was recorded for 4087 singleton pregnancies (20.3%): 4057 live births, 27 intrauterine deaths and 3 neonatal deaths. Compared with pregnancies without a recorded outcome, those with an outcome were examined earlier in the study (median year 2016 vs. 2020), were more often nulliparous (73.2% vs. 60.6% of those with a recorded history) and had a slightly shorter cervix (median 35.0 vs. 36.0 mm, *p* < 0.001; CL < 25 mm 2.3% vs. 1.8%, *p* = 0.04), whereas maternal age (30.3 vs. 30.7 years) and body-mass index (21.8 vs. 22.7 kg/m^2^) were similar (Table S2).

**Figure 3:**
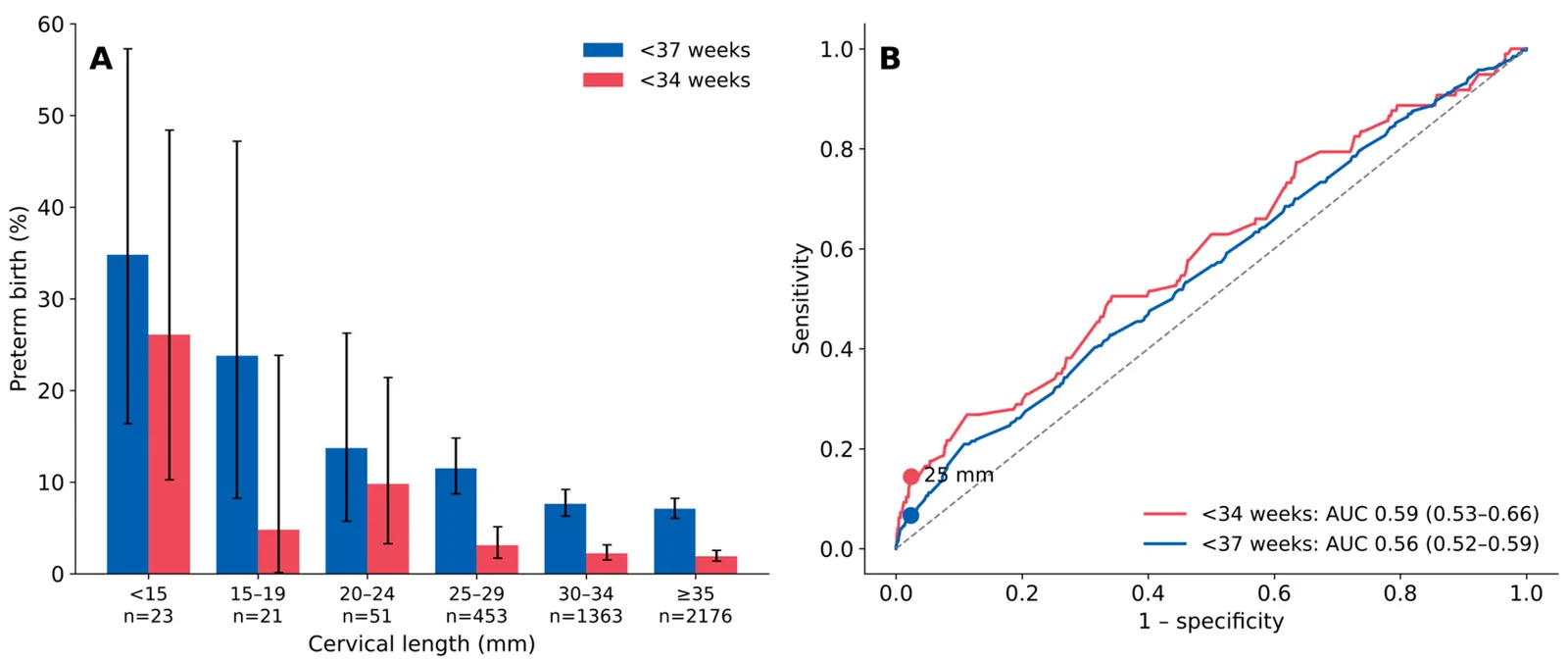
(**A**) Preterm birth before 37 and before 34 weeks (all causes) according to cervical length category in the 4087 pregnancies with a recorded delivery (bars, exact 95% CI); these are proportions within the outcome-ascertained subgroup, not population rates. (**B**) Receiver-operating-characteristic curves of cervical length for birth before 34 and before 37 weeks; the dots mark the 25 mm threshold and the dashed diagonal is the line of no discrimination.

Preterm birth occurred before 37 weeks in 330 pregnancies (8.1%, 95% CI 7.3–9.0), before 34 weeks in 97 (2.4%, 1.9–2.9), before 32 weeks in 54 (1.3%) and before 28 weeks in 19 (0.5%). Rates increased steeply as CL decreased (Table 4; Figure 3A): birth < 34 weeks occurred in 1.9% of women with CL ≥ 35 mm, 2.2% at 30–34.9 mm, 3.1% at 25–29.9 mm, 9.8% at 20–24.9 mm, 4.8% at 15–19.9 mm and 26.1% at <15 mm (*p* for trend = 0.002), and birth < 37 weeks in 7.1%, 7.6%, 11.5%, 13.7%, 23.8% and 34.8% respectively (*p* for trend < 0.001). Women with a CL < 25 mm (*n* = 95) delivered before 37, 34 and 32 weeks in 21.1%, 12.6% and 8.4% of cases, compared with 7.8%, 2.1% and 1.2% of those with CL ≥ 25 mm.

In logistic regression adjusted for maternal age, body-mass index, smoking, IVF, nulliparity, previous late miscarriage or preterm birth and calendar year (complete cases, *n* = 3690; 89 and 296 events), each 5 mm decrease in CL was associated with an OR of 1.47 (95% CI 1.26–1.70) for birth < 34 weeks and 1.27 (1.15–1.39) for birth < 37 weeks; a CL < 25 mm carried an adjusted OR of 9.1 (4.6–17.9) for birth < 34 weeks and 4.0 (2.3–6.7) for birth < 37 weeks (with the binary study period instead of calendar year: 8.3, 4.3–16.3, and 3.6, 2.2–6.1; Table S4). Among the covariates, body-mass index was weakly associated with birth < 37 weeks and IVF conception showed a trend in the same direction; the calendar-year association within this selectively ascertained subgroup should not be read as a population trend in incidence (Section 4.5). Neonatal-unit transfer was recorded too rarely (0 vs. 7 cases) to be analysed. The exploratory neonatal data are shown in Table 5 with their denominators: infants born to women with a CL < 25 mm had a lower median birth weight (3100 vs. 3240 g; recorded in 92/95 and 3894/3992 deliveries), a birth weight < 2500 g in 15/92 (16.3%) versus 255/3894 (6.5%) and <1500 g in 8/92 (8.7%) versus 41/3894 (1.1%); a 1-min Apgar score < 7 was recorded in 4/59 (6.8%) versus 22/2069 (1.1%) of those with an available score, and PPROM in 7/95 (7.4%) versus 61/3992 (1.5%). These comparisons are descriptive and do not support conclusions about clinical benefit.

### 3.5. Screening Performance of Cervical Length Thresholds

Despite the strong association, the sensitivity of any single threshold for preterm birth was low (Table 6; Figure 3B). A CL < 25 mm identified 12 of 97 births < 34 weeks (sensitivity 12.4%, specificity 97.9%, positive predictive value 12.6%, positive likelihood ratio 6.0, relative risk 5.9) and 6.1% of births < 37 weeks; lowering the threshold to 15 mm increased the positive likelihood ratio to 14.5 but reduced sensitivity to 6.2%, and raising it to 30 mm increased sensitivity to 26.8% at the cost of a false-positive rate of 13%. The AUC of CL as a continuous variable was 0.61 (95% CI 0.53–0.68) for birth < 32 weeks, 0.59 (0.53–0.66) for <34 weeks and 0.56 (0.52–0.59) for <37 weeks. Eighty-five of the 97 births before 34 weeks (87.6%, 95% CI 79.4–93.4) and 310 of the 330 births before 37 weeks (93.9%, 90.8–96.3) occurred in women whose index CL was ≥25 mm.

Because CL is a predictor of spontaneous rather than indicated preterm birth, the analysis was repeated with the surrogate definition of “probably spontaneous” preterm birth (Table S5). Of the 97 births < 34 weeks, 32 were elective caesarean sections, 23 emergency caesarean sections and 10 vaginal births (mode unknown in 32); PPROM was recorded in 15; 43 met the surrogate definition of probably spontaneous birth and 27 were elective caesarean sections without any marker of spontaneous onset. Restricting the preterm events to probably spontaneous births increased the sensitivity of CL < 25 mm from 12.4% (6.6–20.6) to 16.3% (7/43; 6.8–30.7), the AUC from 0.59 to 0.62 and the adjusted OR for CL < 25 mm from 9.1 to 14.3 (5.8–35.4); for birth < 37 weeks the corresponding sensitivity rose from 6.1% to 7.9% (11/140). Excluding only the 27 elective caesarean births gave intermediate values (sensitivity 12.9%, AUC 0.60). With the most restrictive definition—documented spontaneous onset of labour or PPROM only—19 births < 34 weeks and 56 births < 37 weeks remained, and CL < 25 mm identified 4/19 (21.1%, 6.1–45.6; AUC 0.63) and 7/56 (12.5%, 5.2–24.1; AUC 0.57). The direction of these changes is as expected, but the sensitivity of a single mid-trimester CL for surrogate-defined probable spontaneous early preterm birth remained low, with wide confidence intervals in the restricted analyses.

The discrimination of the stored FMF risk for spontaneous birth < 34 weeks against the all-cause endpoint (AUC 0.56, 0.49–0.63, in 3641 pregnancies; CL alone 0.57, 0.50–0.65) is given in Table S8 for completeness; as the two outcomes differ, this comparison does not evaluate the algorithm and is not interpreted further.

### 3.6. Outcome in Women with a Short Cervix According to Counselling

Among the 95 women with a CL < 25 mm and a recorded delivery, 69 had an explicit progesterone mention (median CL 19.0 mm) and 26 had not (median CL 22.0 mm). Birth before 37 weeks occurred in 15 (21.7%) and 5 (19.2%), and before 34 weeks in 9 (13.0%) and 3 (11.5%), respectively. Given the difference in CL between the groups, the small numbers and the absence of information on actual treatment, these figures are reported for completeness only and do not permit any inference on treatment effect.

## 4. Discussion

### 4.1. Principal Findings

In more than 20,000 singleton pregnancies from a routine-screening population examined over 15 years with a two-step transabdominal–transvaginal protocol, a CL < 25 mm was found in 1.9% and <15 mm in 0.6%, and a previous late miscarriage or preterm birth increased the prevalence of a short cervix about five-fold compared with nulliparous women and seven-fold compared with a previous term birth. Within the outcome-ascertained subgroup, a short cervix carried a nine-fold adjusted odds of birth before 34 weeks and an eight-fold excess of very-low-birth-weight infants (8/92 vs. 41/3894), yet 88% (95% CI 79–93%) of births before 34 weeks occurred in women with a normal CL. The structured ultrasound report captured the migration of documented counselling to the full ≤25 mm range: before 2017 progesterone was mentioned for most women with a CL below about 22 mm but for fewer than a quarter of those with 22.5–24.9 mm, whereas from 2017 onwards three-quarters of women with a CL of 20–24.9 mm had a documented recommendation, without any increase below 20 mm. A data-driven change-point search placed the shift in 2017–2019, as a level change rather than a trend, and the shift was not explained by the measured composition of the population.

### 4.2. Prevalence and Distribution in Context

The prevalence of a short cervix in our population is at the lower end of the range reported by universal screening programmes: 0.8–1% for CL ≤ 20 mm and about 2% for ≤25 mm in the low-risk populations of Orzechowski et al. and Temming et al. [18,19], 1.5–1.7% for ≤15 mm in the FMF series [4,5,7] and 2.3% for 10–20 mm in the PREGNANT screening population [8]. Our 0.6% for <15 mm and 1.0% for <20 mm are somewhat lower, which is compatible with a population with a low background preterm birth rate, with the exclusion of multiple pregnancies, and possibly with a slight loss of borderline short cervices in a protocol that starts transabdominally. The strong association with obstetric history reproduces the FMF model [6] and supports the continued use of history-based risk stratification alongside CL, while the benefit of progesterone itself appears confined to women whose cervix is actually short [34].

The shape of the CL distribution deserves comment. The discontinuity at 30 mm and the shortfall of values between 25 and 30 mm are the signature of the two-step protocol: transabdominal measurement systematically overestimates CL [25,26,27,28], so that a transabdominal value of 30–34 mm is often a transvaginal value of 25–29 mm. The practical consequence is that our estimates of the distribution above 30 mm are transabdominal and not directly comparable with transvaginal reference ranges, whereas every short cervix below 25 mm was confirmed transvaginally under standardised conditions. The 30 mm trigger is lower than the 35–36 mm safety margin proposed by Friedman et al. [26] and endorsed by the SMFM [13], so that some truly short cervices may have been missed. Among the 6441 pregnancies with an index CL of 30–34.9 mm, 254 underwent a further cervical-length assessment within the 16–24-week window, of whom 10 (3.9%, 95% CI 1.9–7.1) subsequently had a measurement < 25 mm (Table S9). Repeat examinations were not scheduled systematically by our centre; most occurred following referral or patient-initiated reassessment, frequently because the attending obstetrician had raised concern about cervical shortening (the reason for re-attendance is not recorded in the database, so this is a description of the centre’s practice rather than a measured variable). Consequently, this subgroup was clinically selected and cannot be considered representative of all pregnancies with an initial CL of 30–34.9 mm. Furthermore, subsequent cervical shortening cannot be distinguished from a short cervix missed at the index examination. These observations therefore demonstrate the possibility of later identification of a short cervix but do not permit estimation of the false-negative rate of the initial transabdominal screening protocol. For context, the rate of birth < 34 weeks in the 30–34.9 mm band (2.2%, 1.5–3.1) was only marginally higher than at ≥35 mm (1.9%, 1.4–2.5) and lower than at 25–29.9 mm (3.1%); although these rates do not suggest a marked excess of preterm birth in the 30–34.9 mm band, they cannot establish the number of short cervices missed by the initial screening protocol. The published transabdominal overestimation of 2–5 mm [25,26,27,28] would move cervices from the 30–34.9 mm band mainly to the 25–29.9 mm band, where management is not altered. We regard the under-ascertainment of treatable short cervices as a real limitation whose size cannot be quantified retrospectively, and we now recommend the 35 mm transabdominal trigger (Section 4.6), without claiming that our data show how many cases this change would recover.

### 4.3. Association with Preterm Birth and Screening Performance

The gradient of preterm birth across CL categories closely reproduces the NICHD data [3]: a relative risk of about 6 for birth < 34 weeks at the 25 mm threshold and of 12 at 15 mm, with rates of 26% and 35% for birth < 34 and <37 weeks in the shortest category. The adjusted OR of about 9 for CL < 25 mm and the excess of very-low-birth-weight infants and PPROM describe, for the first time in a Romanian population, the neonatal burden concentrated in this small group, within the limits of an outcome-ascertained subgroup and of exploratory neonatal data. At the same time, the AUC of 0.59 for birth < 34 weeks and the sensitivity of 12% at the 25 mm threshold demonstrate the distinction between individual risk and population-level detection. A short cervix identifies a small group at markedly increased risk, but most preterm births arise outside this phenotype. This is consistent with the nuMoM2b cohort, in which a cervical length ≤ 25 mm at 16–22 weeks identified only 8% of subsequent spontaneous preterm births among nulliparous women [24], and it explains why universal screening changes population preterm-birth rates by only a few percent in pre/post studies [16,17,18,19,20,21]. Our endpoint was broader and included all preterm births, irrespective of spontaneous or medically indicated onset; inclusion of preterm births not mediated by cervical shortening would be expected to reduce the sensitivity of cervical length, and the sensitivity analysis with surrogate-defined probable spontaneous birth confirmed the direction of this effect (sensitivity 16% and AUC 0.62 for birth < 34 weeks; 21% with the most restrictive definition, based on 19 events) without changing its order of magnitude. Thus, the low population sensitivity observed here should not be interpreted as evidence against cervical-length screening or progesterone treatment [10,11], but rather as a limitation of cervical shortening as a phenotype of the overall preterm-birth syndrome.

We have not attempted to evaluate the FMF risk algorithm, whose target is spontaneous preterm birth, against our all-cause endpoint; the figures are retained in the Supplementary Materials only to document that they were examined. Clinically, the distinction matters because the risk figure given to a woman with a normal cervix refers to spontaneous preterm birth and may understate her overall probability of preterm delivery.

### 4.4. Real-Time Documentation of a Change in Practice

To our knowledge this is the first study to reconstruct the adoption of a treatment threshold from the contemporaneous text of ultrasound reports. Three features of the result are of interest. First, below 20 mm counselling was already high in 2011–2016, reflecting the FMF trial [7] and the PREGNANT trial [8], and did not change; the entire shift occurred in the 20–25 mm band, exactly the range over which the updated IPD meta-analysis [10], building on the 2012 analysis [9], consolidated the evidence and the subsequent guidelines [13,14,15] endorsed treatment across the whole range. Second, before 2017 the de facto threshold was not a round 20 mm but about 22 mm, with a steep decline in counselling between 22 and 25 mm; this “soft” threshold probably reflects the clinicians’ awareness of measurement variability and of the 2011 trial’s inclusion range. Third, the change was located by the data themselves, not by assumption: the change-point search selected 2017, with 2018 and 2019 practically equivalent, and 81% of clustered bootstrap samples placed the change in 2017–2019; the interrupted-time-series model showed a level shift without preceding or subsequent trend. We therefore describe a window rather than a single year of change. This window begins the year the updated IPD meta-analysis appeared online and ends one to two years after the guideline statements, which is a plausible diffusion time for new evidence in a single specialised unit. We emphasise that this is a temporal coincidence in an observational series: the analysis cannot attribute the change to a specific publication, and the annual numbers (5–21 pregnancies) are small. The change was also not explained by the measured composition of the population, which shifted only modestly between periods (Table S7). The sensitivity analysis with the broader definition of documented management shows that the apparent decline after 2023 was an artefact of a change in report wording rather than of practice, and underlines a general caveat of text-derived process measures: they measure documentation, and documentation templates evolve.

The counselling rates themselves—two-thirds to three-quarters of women with a short cervix—also merit reflection. They are similar to the proportion of eligible women who received progesterone in implementation studies [17,19] and reflect real-world limits: women already on progesterone for other indications, women referred back to their obstetrician for management, emergency cerclage counselling in the shortest cervices, and incomplete transcription. Because the report documents a recommendation and not a dispensing record, the true treatment rate cannot be derived from our data.

### 4.5. Strengths and Limitations

The strengths of this study are its size—a large, long-term CL screening cohort from Central and Eastern Europe—, the largely unselected routine-screening population, the standardised transvaginal confirmation of every short cervix, the 15-year span that encompasses the change in evidence, and the availability of the original counselling text for every examination. The limitations are substantial and should be weighed carefully. Delivery outcome was known for only one-fifth of pregnancies; the pregnancies with a recorded outcome were examined earlier in the study period, were more often nulliparous and had a slightly shorter cervix than those without (Table S2), so that absolute preterm-birth rates cannot be extrapolated as population prevalence and the gradient across CL categories, while robust, is based on small numbers in the shortest categories. Accordingly, all outcome analyses, including the neonatal comparisons, should be interpreted as estimates of association within the outcome-ascertained subgroup rather than as population-level estimates of incidence, and the neonatal findings are exploratory and cannot support conclusions about clinical benefit. The onset of labour was rarely recorded, so that spontaneous and indicated preterm births could be separated only through surrogates that are not a validated spontaneous endpoint; the screening performance we report for the all-cause endpoint understates that for spontaneous preterm birth, as the sensitivity analysis indicates. The division of the study period at 2017 was made with knowledge of the data and of the evidence timeline; we have therefore complemented it with a data-driven change-point analysis, but the location of the change remains an estimate with a three-year window, and the small annual numbers limit any finer resolution. Counselling was extracted from templated text and reflects recommendation rather than treatment; the effect of progesterone cannot be assessed, and the comparison of outcomes between counselled and non-counselled women is confounded by indication. The two-step measurement protocol with a 30 mm rather than 35 mm transabdominal trigger means that the distribution above 30 mm is transabdominal and that an unknown number of short cervices may have been missed; the repeat measurements available in a clinically selected subgroup (Section 4.2) do not permit a retrospective estimate of the false-negative rate. The keyword-based extraction of counselling depends on the wording of the report, which changed in 2023; the repeated extraction for this revision also identified a few mentions missed by the original search, and both indicators are reported. Finally, the study period included the introduction of new ultrasound systems and additional operators, and multiple examinations of the same woman were reduced to one index scan.

### 4.6. Clinical Implications

For clinicians in settings similar to ours, the data support three messages. Universal mid-trimester CL assessment, even when started transabdominally, identifies the roughly 2% of women whose risk of early preterm birth is increased about nine-fold and who are eligible for vaginal progesterone under current guidelines; when the transabdominal route is used first, the 35 mm trigger for transvaginal measurement recommended by the SMFM and ISUOG should be preferred to the 30 mm trigger used in our unit, which we have now changed; women with a previous late miscarriage or preterm birth deserve particular attention, since one in ten has a short cervix. A normal mid-trimester cervix, however, should not be interpreted as reassurance against preterm birth: in our outcome-ascertained subgroup, 88% (95% CI 79–93%) of births before 34 weeks occurred after an index CL ≥ 25 mm. Cervical-length screening should therefore be understood as identifying a small, clinically actionable high-risk phenotype rather than as a population-wide rule-out test for preterm birth, and neither the CL nor the calculated risk should be presented to women as such. Lastly, structured, template-based reporting makes it possible to audit the translation of evidence into practice at the level of the individual consultation; the 20–25 mm band, where our practice changed between 2017 and 2019, is a natural indicator for such audits in other centres.

## 5. Conclusions

In a largely unselected Romanian routine-screening population, a mid-trimester CL < 25 mm was found in 1.9% of singleton pregnancies and, within the one-fifth with a recorded delivery, was associated with nine-fold adjusted odds of birth before 34 weeks and an exploratory excess of very-low-birth-weight infants, while still accounting for only one in eight early preterm births. The structured ultrasound report documented, in real time, the migration of the explicit vaginal progesterone recommendation from a de facto threshold of about 22 mm to the full ≤25 mm range endorsed by the updated evidence and guidelines; a data-driven change-point analysis located this shift in 2017–2019, independent of population composition. Routine CL screening in this setting identifies a small, high-risk group amenable to evidence-based preventive intervention rather than the majority of women who will subsequently deliver preterm, and its documentation can serve as an audit tool for guideline adoption.

## Figures and Tables

**Table 1:** Characteristics of the study population (*n* = 20,168 singleton pregnancies).

Characteristic	Value
Women, *n*	17,966
Year of index scan, range	2011–2026
Gestational age at index scan, weeks, median (IQR)	21.6 (20.7–22.3)
Index scan at 18^+0^–24^+6^ weeks, *n* (%)	19,366 (96.0)
Maternal age, years, median	30.6
Body-mass index, kg/m^2^, median	22.5
Nulliparous, *n* (%) ^a^	11,273 (63.3)
Previous spontaneous delivery at 16–36 weeks (late miscarriage or preterm birth), *n* (%) ^a^	471 (2.6)
Conception by IVF, *n* (%)	730 (3.6)
Smoking, *n* (%)	3286 (16.3)
Cervical length, mm, mean ± SD	36.2 ± 6.5
Cervical length, mm, median (IQR)	35.8 (31.9–40.0)
Funnelling assessed, *n*; positive, *n* (%)	7219; 76 (1.1)
Delivery outcome recorded, *n* (%)	4087 (20.3)

^a^ Denominator 17,817 pregnancies with recorded obstetric history. IQR, interquartile range; IVF, in vitro fertilisation.

**Table 2:** Cervical length according to obstetric history (categories of the FMF preterm-birth algorithm; deliveries at 16–23 weeks correspond to late miscarriage).

Obstetric History	*n*	Median CL, mm	CL < 25 mm, *n* (%)
Nulliparous	11,273	35.0	223 (2.0)
Previous birth after 36 weeks	6073	37.3	82 (1.4)
Previous preterm birth at 34–36 weeks	173	34.9	16 (9.2)
Previous preterm birth at 24–33 weeks	111	34.5	11 (9.9)
Previous late miscarriage at 16–23 weeks	187	33.8	18 (9.6)

**Table 3:** Documented counselling at the index scan according to cervical length and study period (explicit progesterone mention; broader ‘documented management’ indicator; cerclage and pessary discussed as options).

CL Category	Period	*n*	Progesterone Mentioned, *n* (%)	Documented Management, *n* (%)	Cerclage Discussed, *n* (%)	Pessary Discussed, *n* (%)
<15 mm	2011–2016	37	31 (83.8)	31 (83.8)	31 (83.8)	0 (0.0)
	2017–2026	84	50 (59.5)	50 (59.5)	59 (70.2)	4 (4.8)
15–19.9 mm	2011–2016	39	36 (92.3)	36 (92.3)	30 (76.9)	0 (0.0)
	2017–2026	45	35 (77.8)	37 (82.2)	34 (75.6)	2 (4.4)
20–24.9 mm	2011–2016	63	31 (49.2)	31 (49.2)	22 (34.9)	0 (0.0)
	2017–2026	120	88 (73.3)	101 (84.2)	76 (63.3)	3 (2.5)
25–29.9 mm	2011–2016	609	6 (1.0)	6 (1.0)	2 (0.3)	0 (0.0)
	2017–2026	1412	49 (3.5)	54 (3.8)	25 (1.8)	4 (0.3)
30–34.9 mm	2011–2016	1938	3 (0.2)	3 (0.2)	3 (0.2)	0 (0.0)
	2017–2026	4503	19 (0.4)	19 (0.4)	6 (0.1)	0 (0.0)
≥35 mm	2011–2016	2704	2 (0.1)	2 (0.1)	2 (0.1)	0 (0.0)
	2017–2026	8614	9 (0.1)	9 (0.1)	9 (0.1)	0 (0.0)

Documented management = explicit progesterone mention, or generic prescription wording in a cervical-reassessment context, or cerclage, or pessary (see Section 2.3). Mentions describe the content of the counselling letter and are not verified prescriptions or procedures.

**Table 4:** Preterm birth and neonatal outcome according to cervical length at the index scan (*n* = 4087 pregnancies with recorded delivery).

CL Category	*n*	<37 Weeks, *n* (%)	<34 Weeks, *n* (%)	<32 Weeks, *n* (%)	<28 Weeks, *n*	GA at Delivery, Weeks, Median	Birth Weight, g, Median
<15 mm	23	8 (34.8)	6 (26.1)	5 (21.7)	2	37.9	2965
15–19.9 mm	21	5 (23.8)	1 (4.8)	1 (4.8)	0	38.3	3120
20–24.9 mm	51	7 (13.7)	5 (9.8)	2 (3.9)	2	38.7	3100
25–29.9 mm	453	52 (11.5)	14 (3.1)	7 (1.5)	4	38.9	3190
30–34.9 mm	1363	104 (7.6)	30 (2.2)	16 (1.2)	4	38.9	3210
≥35 mm	2176	154 (7.1)	41 (1.9)	23 (1.1)	7	39.0	3260
All	4087	330 (8.1)	97 (2.4)	54 (1.3)	19	38.9	3240

GA, gestational age.

**Table 5:** Exploratory neonatal outcomes according to cervical length < 25 mm versus ≥25 mm (*n*/N with the number of deliveries in which each item was recorded).

Outcome	CL < 25 mm (*n* = 95)	CL ≥ 25 mm (*n* = 3992)
Gestational age at delivery, weeks, median	38.3	38.9
Birth < 37 weeks, *n* (%)	20 (21.1)	310 (7.8)
Birth < 34 weeks, *n* (%)	12 (12.6)	85 (2.1)
Birth < 32 weeks, *n* (%)	8 (8.4)	46 (1.2)
Birth weight, g, median (*n* available)	3100 (92)	3240 (3894)
Birth weight < 2500 g, *n* (% of recorded)	15 (16.3)	255 (6.5)
Birth weight < 1500 g, *n* (% of recorded)	8 (8.7)	41 (1.1)
Apgar 1 min < 7, *n*/*n* available (%)	4/59 (6.8)	22/2069 (1.1)
PPROM recorded, *n* (%)	7 (7.4)	61 (1.5)
Caesarean delivery, *n*/*n* with known mode (%)	63/87 (72.4)	3037/3845 (79.0)

PPROM, preterm prelabour rupture of membranes.

**Table 6:** Screening performance of cervical length thresholds for preterm birth (*n* = 4087).

Threshold	Outcome	Sensitivity, %	Specificity, %	PPV, %	NPV, %	LR+	LR−	RR
<15 mm	<34 weeks	6.2	99.6	26.1	97.8	14.5	0.94	11.7
<20 mm	<34 weeks	7.2	99.1	15.9	97.8	7.8	0.94	7.2
<25 mm	<34 weeks	12.4	97.9	12.6	97.9	6.0	0.90	5.9
<30 mm	<34 weeks	26.8	86.9	4.7	98.0	2.1	0.84	2.4
<15 mm	<37 weeks	2.4	99.6	34.8	92.1	6.1	0.98	4.4
<25 mm	<37 weeks	6.1	98.0	21.1	92.2	3.0	0.96	2.7
<30 mm	<37 weeks	21.8	87.3	13.1	92.7	1.7	0.90	1.8

PPV/NPV, positive/negative predictive value; LR, likelihood ratio; RR, relative risk.

## Data Availability

The pseudonymised dataset is held by the Maternal-Fetal Medicine Center, Timișoara, and is not publicly available because of patient-confidentiality restrictions; aggregate data are available from the corresponding author on reasonable request.

## Supplementary Materials

The following supporting information can be downloaded at https://www.mdpi.com/article/10.3390/jcm15218310/s1.
