# Supplementary material for: Mid-Trimester Cervical Length Screening in Routine Practice: Short-Cervix Prevalence, Preterm Birth and the Shift of the Progesterone Counselling Threshold from 20 to 25 mm in a 15-Year Single-Centre Cohort of 20,168 Singleton Pregnancies
Source: J Clin Med. 2026 Oct 25;15(21):8310. doi: 10.3390/jcm15218310 (PMC13764427; doi:10.3390/jcm15218310)
Supplement: Supplementary file 1 [file jcm-15-08310-s001.zip › jcm-4586164-supplementary.pdf]

Supplementary Materials

Mid-Trimester Cervical Length Screening in Routine Practice: Short-Cervix Prevalence, Preterm Birth and the Shift of the Progesterone Counselling Threshold from 20 to 25 mm in a 15-Year Single-Centre Cohort of 20,168 Singleton Pregnancies

Maria Cezara Mureșan, Marius Bogdan Mureșan, Dan Dumitrașcu Biriș, Daniela-Eugenia Popescu

Journal of Clinical Medicine — Supplementary Materials

Figure S1

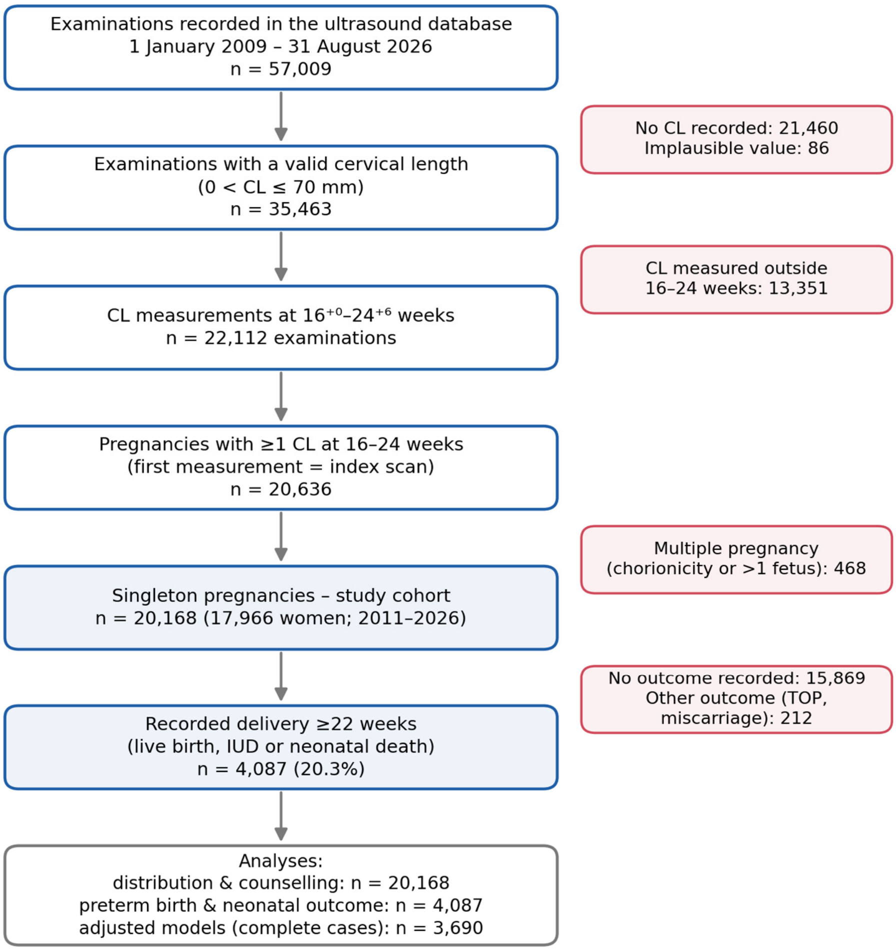

Figure S1. Flow of examinations and pregnancies through the study. CL, cervical length; IUD, intrauterine death; TOP, termination of pregnancy. Examinations belonging to the same woman were grouped into pregnancies using the patient identifier and the examination dates and gestational ages; the index scan was the first CL measurement at 16^{+0}–24^{+6} weeks of each pregnancy.

Table S1

Table S1. Annual number of index scans, prevalence of a short cervix and completeness of delivery outcome, 2011–2026 (singleton pregnancies, n = 20,168).

Year of index scan	Index scans, n	CL <25 mm, n (%)	CL <15 mm, n	Progesterone counselling at CL <25 mm, n	Delivery outcome recorded, n (%)
2011	497	22 (4.43)	5	13	280 (56.3)
2012	619	26 (4.20)	7	21	337 (54.4)
2013	654	21 (3.21)	8	15	279 (42.7)
2014	996	19 (1.91)	3	12	391 (39.3)
2015	1,220	19 (1.56)	5	15	438 (35.9)
2016	1,404	32 (2.28)	9	22	328 (23.4)
2017	1,491	37 (2.48)	15	25	484 (32.5)
2018	1,498	21 (1.40)	7	16	240 (16.0)
2019	1,639	30 (1.83)	13	24	239 (14.6)
2020	1,496	25 (1.67)	9	17	170 (11.4)
2021	1,644	36 (2.19)	14	29	189 (11.5)
2022	1,753	25 (1.43)	9	21	220 (12.5)
2023	1,364	11 (0.81)	3	9	250 (18.3)
2024	1,287	33 (2.56)	7	11	132 (10.3)
2025	1,573	19 (1.21)	4	10	86 (5.5)
2026 (to August)	1,033	12 (1.16)	3	11	24 (2.3)
**Total**	**20,168**	**388 (1.92)**	**121**	**271**	**4,087 (20.3)**

CL, cervical length at the index scan. Progesterone counselling refers to an explicit mention of vaginal progesterone in the report text (primary indicator, repeated extraction; see Section 2.3 of the main text); the lower counts in 2024–2025 reflect the change in report wording.

Table S2

Table S2. Characteristics of singleton pregnancies with and without a recorded delivery outcome.

Characteristic	Delivery outcome recorded (n = 4,087)	No delivery outcome (n = 16,081)	p
Year of index scan, median	2016	2020	—
Gestational age at index scan, weeks, median	21.4	21.6	<0.001
Maternal age, years, median	30.3	30.7	<0.001
Body-mass index, kg/m^2, median	21.8	22.7	<0.001
Nulliparous, n (% of recorded history) [a]	2,749 (73.2)	8,524 (60.6)	<0.001
Previous spontaneous delivery at 16–36 weeks (late miscarriage or preterm birth), n (% of recorded history) [a]	81 (2.2)	390 (2.8)	0.04
Conception by IVF, n (%)	184 (4.5)	546 (3.4)	<0.001
Smoking, n (%)	484 (11.8)	2,802 (17.4)	<0.001
Cervical length, mm, median (IQR)	35.0 (31.0–40.0)	36.0 (32.0–40.0)	<0.001
Cervical length <30 mm, n (%)	548 (13.4)	1,861 (11.6)	0.001
Cervical length <25 mm, n (%)	95 (2.32)	293 (1.82)	0.04
Progesterone counselling documented, n (%)	82 (2.0)	277 (1.7)	0.25

[a] Obstetric history recorded in 3,757 and 14,060 pregnancies, respectively. p values from the Mann–Whitney U test (continuous variables) or the χ^2 / Fisher exact test (categorical variables). IQR, interquartile range; IVF, in-vitro fertilisation. Pregnancies with a recorded outcome were examined earlier in the study period, were more often nulliparous and had a slightly shorter cervix; outcome analyses in the main text are therefore interpreted as estimates of association within the outcome-ascertained subgroup rather than as population estimates of preterm-birth incidence.

Table S3

Table S3. Annual documented progesterone counselling at a cervical length of 20–24.9 mm (n = 183 pregnancies): explicit progesterone mention (primary indicator) and broader “documented management” indicator.

Year	n	Explicit mention, n (%)	Exact 95% CI, %	Documented management, n (%)	3-year moving average of explicit mention, %
2011	9	4 (44.4)	13.7–78.8	4 (44.4)	–
2012	12	8 (66.7)	34.9–90.1	8 (66.7)	56.7
2013	9	5 (55.6)	21.2–86.3	5 (55.6)	57.6
2014	12	6 (50.0)	21.1–78.9	6 (50.0)	53.3
2015	9	5 (55.6)	21.2–86.3	5 (55.6)	42.4
2016	12	3 (25.0)	5.5–57.2	3 (25.0)	48.6
2017	14	9 (64.3)	35.1–87.2	9 (64.3)	51.4
2018	9	6 (66.7)	29.9–92.5	6 (66.7)	72.2
2019	13	11 (84.6)	54.6–98.1	11 (84.6)	80.0
2020	13	11 (84.6)	54.6–98.1	11 (84.6)	84.6
2021	13	11 (84.6)	54.6–98.1	11 (84.6)	89.5
2022	12	12 (100.0)	73.5–100.0	12 (100.0)	93.9
2023	8	8 (100.0)	63.1–100.0	8 (100.0)	68.3
2024	21	8 (38.1)	18.1–61.6	19 (90.5)	56.1
2025	12	7 (58.3)	27.7–84.8	9 (75.0)	52.6
2026	5	5 (100.0)	47.8–100.0	5 (100.0)	–

Documented management = explicit progesterone mention, or generic prescription wording (“treatment according to the prescription”) in the context of cervical re-assessment advice, or cerclage, or pessary; it is not a verified prescription rate. The two indicator columns overlap and must not be added. Annual denominators are small (5–21). The generic prescription wording appeared in the report template from 2023 (12 reports in 2024, 2 in 2025, 1 in 2026 at 20–24.9 mm). 2026 covers January–August. The moving average is centred and unweighted.

Table S4

Table S4. Adjusted logistic regression models for preterm birth according to cervical length, with calendar year (continuous; main text) or binary study period (2011–2016 vs 2017–2026; originally submitted version) as the time covariate.

Outcome	CL exposure	n (complete cases)	Events	OR (95% CI), calendar year	OR (95% CI), binary period
<34 weeks	Per 5 mm decrease in CL	3690	89	1.47 (1.26–1.70)	1.44 (1.24–1.67)
<34 weeks	CL <25 vs ≥25 mm	3690	89	9.06 (4.59–17.88)	8.33 (4.25–16.30)
<37 weeks	Per 5 mm decrease in CL	3690	296	1.27 (1.15–1.39)	1.24 (1.13–1.36)
<37 weeks	CL <25 vs ≥25 mm	3690	296	3.96 (2.34–6.71)	3.64 (2.16–6.13)

Each cell is a separate complete-case model adjusted for maternal age, body-mass index, smoking, IVF conception, nulliparity, previous spontaneous delivery at 16–36 weeks and the time covariate; continuous CL and the binary short-cervix exposure are fitted in separate models. All $p < 0.001$. Odds ratios overestimate relative risks when the outcome is not rare (birth <37 weeks). Adjustment for time does not correct the differential ascertainment of outcome (Table S2).

Table S5

Table S5. Sensitivity analysis restricting preterm births to surrogate-defined probable spontaneous births: screening performance of a cervical length <25 mm.

Definition of the preterm endpoint	n analysed	Events	Sensitivity, n/N (%; 95% CI)	Specificity, %	PPV, %	AUC of CL
<34 weeks, all preterm births	4087	97	12/97 (12.4; 6.6–20.6)	97.9	12.6	0.59
<34 weeks, excluding elective caesarean preterm births without markers of spontaneous onset (n = 27)	4060	70	9/70 (12.9; 6.1–23.0)	97.9	9.8	0.60
<34 weeks, surrogate-defined probable spontaneous preterm	4033	43	7/43 (16.3; 6.8–30.7)	97.9	7.8	0.62

Definition of the preterm endpoint	n analysed	Events	Sensitivity, n/N (%; 95% CI)	Specificity, %	PPV, %	AUC of CL
births only (n = 43)						
<34 weeks, documented spontaneous onset of labour or PPROM only (n = 19)	4009	19	4/19 (21.1; 6.1–45.6)	97.9	4.6	0.63
<37 weeks, all preterm births	4087	330	20/330 (6.1; 3.7–9.2)	98.0	21.1	0.56
<37 weeks, excluding elective caesarean preterm births without markers (n = 147)	3940	183	13/183 (7.1; 3.8–11.8)	98.0	14.8	0.56
<37 weeks, surrogate-defined probable spontaneous preterm births only (n = 140)	3897	140	11/140 (7.9; 4.0–13.6)	98.0	12.8	0.55
<37 weeks, documented spontaneous onset of labour or PPROM only (n = 56)	3813	56	7/56 (12.5; 5.2–24.1)	98.0	8.5	0.57

Surrogate definition: a preterm delivery was classified as probably spontaneous when the mode of delivery was vaginal, vacuum or emergency caesarean section, or when PPROM or spontaneous onset of labour was recorded; as probably indicated when it was an elective caesarean section without any of these features; deliveries with unknown mode of delivery were retained only in the all-cause endpoint. Births <34 weeks (n = 97): elective caesarean 32, emergency caesarean 23, vaginal 10, mode unknown 32; PPROM recorded in 15; probably spontaneous 43; elective caesarean without markers 27. Births <37 weeks (n = 330): elective caesarean 160, emergency caesarean 63, vaginal 57, unknown 50; PPROM 40; probably spontaneous 140; elective without markers 147. In the restricted (probably spontaneous) analyses the adjusted OR for CL <25 mm (same covariates as Table S4, calendar year) was 14.3 (5.8–35.5) for birth <34 weeks (n = 3639) and 5.6 (2.8–11.2) for birth <37 weeks (n = 3518). The surrogate is not a validated spontaneous endpoint: an emergency caesarean section may be performed for pre-eclampsia, placental abruption or fetal compromise without spontaneous onset of labour, and an elective caesarean section may follow a spontaneous onset that was not recorded; the most restrictive definition (documented spontaneous onset or

PPROM) is limited by the rarity of these entries (onset of labour recorded in <10% of deliveries). PPV, positive predictive value; AUC, area under the ROC curve.

Table S6

Table S6. Data-driven location of the change in documented progesterone counselling at a cervical length of 20–24.9 mm (183 pregnancies in 182 women). (A) Likelihood profile over candidate change years and clustered bootstrap selection frequencies; (B) interrupted time series, alternative cut-points and composition-adjusted step models.

(A)

First year of the later period	Δ deviance, explicit indicator	Δ deviance, documented management	Bootstrap selection, explicit, %	Bootstrap selection, documented management, %
2013	9.77	26.71	0.2	0.0
2014	9.33	25.18	0.4	0.0
2015	7.94	21.48	0.2	0.0
2016	7.28	19.24	0.1	0.1
2017	0.00	4.78	38.0	16.6
2018	0.43	1.98	22.4	24.1
2019	0.84	0.00	21.1	41.9
2020	5.07	4.77	1.4	9.4
2021	8.01	8.41	0.1	4.2
2022	9.82	11.14	0.0	3.6
2023	9.94	19.10	0.0	0.0
2024	7.26	23.38	16.1	0.0

(B)

Model	OR (95% CI)	p
Interrupted time series (explicit indicator), pre-2017 linear trend, OR per year	0.82 (0.61–1.11)	0.20
Interrupted time series, level change at 2017	7.64 (1.91–30.58)	0.004
Interrupted time series, change of slope after 2017, OR per year	1.13 (0.81–	0.48

Model	OR (95% CI)	p
	1.57)	
Simple step, 2016 as first year of the later period	1.82 (0.94–3.54)	0.076
Simple step, 2017	2.84 (1.50–5.38)	0.0014
Simple step, 2018	2.71 (1.45–5.06)	0.0018
Simple step, 2019	2.64 (1.41–4.95)	0.0023
Step at 2017 adjusted for maternal age, nulliparity, previous spontaneous delivery at 16–36 weeks, CL and gestational age at the index scan (n = 166 complete cases)	5.01 (2.30–10.90)	<0.001
Same model with linear calendar year added (n = 166)	7.61 (1.90–30.51)	0.004

(A) A single-step binomial model (two proportions, before/from the candidate year) was fitted for each candidate first year of the later period from 2013 to 2024; deviance differences are relative to the best candidate within each indicator and are not likelihood-ratio p values. The best year was 2017 for the explicit indicator and 2019 for the broader indicator. Women (with all their pregnancies) were resampled with replacement 2,000 times (seed 20261017); the selected year fell within 2017–2019 in 81.4% (explicit) and 82.6% (documented management) of resamples, and within 2017–2020 in 82.8% and 92.0%. The 16% of explicit-indicator resamples selecting 2024 reflect the drop of the explicit mention in 2024–2025 caused by the change of template wording. These are stability frequencies under resampling and the specified candidate set, not confidence coverage for a protocol date. (B) The year 2017 was selected after observing the data; the confidence intervals condition on this selection and ignore the search. In the adjusted models the only covariate associated with documentation was CL (OR 0.68 per mm, 0.53–0.87; shorter cervix, more documentation); nulliparity (2.3, 0.98–5.5) and a previous spontaneous delivery at 16–36 weeks (3.4, 0.8–14.7) were imprecisely estimated and calendar year was not associated (0.94 per year, 0.81–1.10) once the step was included. No effect of a specific publication or of progesterone treatment is estimated.

Table S7

Table S7. Composition of the whole cohort and of the 20–24.9 mm band in the two study periods.

Characteristic	2011–2016	2017–2026
All singleton pregnancies, n	5390	14778
Maternal age, years, median	30.1	30.7
Body-mass index, kg/m^2, median	21.6	22.8
Nulliparous, % of recorded history	67.6	61.6
Previous spontaneous delivery at 16–36 weeks, % of recorded history	2.5	2.7
IVF conception, %	2.6	4.0
Smoking, %	13.5	17.3
Cervical length, mm, median	35.0	36.0
Cervical length <25 mm, %	2.58	1.68
**Pregnancies with CL 20–24.9 mm, n**	63	120
Maternal age, years, median	28.7	30.6
Nulliparous, % of recorded history	71	62
Previous spontaneous delivery at 16–36 weeks, %	15	7
IVF conception, %	0	5
Cervical length, mm, median	22.0	22.4

The population became slightly older, heavier and more often smoking and IVF-conceived over time, and the prevalence of a short cervix fell after the first years (Table S1); within the 20–24.9 mm band the differences between periods were modest and the direction of the composition change (fewer women with a previous preterm birth after 2017) would, if anything, be expected to reduce rather than increase documented counselling. The adjusted step models (Table S6B) account for these measured variables; unmeasured changes (operators, template, referral pattern) cannot be excluded.

Table S8

Table S8. Discrimination of the stored FMF patient-specific risk of spontaneous birth <34 weeks and of cervical length alone against the all-cause endpoint of birth <34 weeks (pregnancies with a stored FMF risk and a recorded delivery, n = 3,641).

Predictor	n	Events (all-cause birth <34 weeks)	AUC (95% CI)

Predictor	n	Events (all-cause birth <34 weeks)	AUC (95% CI)
FMF risk of spontaneous birth <34 weeks (CL + obstetric history)	3641	86	0.56 (0.49–0.63)
Cervical length alone (same pregnancies)	3641	86	0.57 (0.50–0.65)

Retained for completeness only. The FMF algorithm targets spontaneous preterm birth, whereas our endpoint includes medically indicated preterm births, whose onset was not recorded; the comparison therefore does not evaluate the algorithm and is not interpreted in the main text.

Table S9

Table S9. Repeat cervical-length measurements in pregnancies with an index CL of 30–34.9 mm: exploratory assessment of the 30 mm (instead of 35 mm) transabdominal trigger for transvaginal measurement.

Item	n	Proportion (95% CI)
Pregnancies with index CL 30–34.9 mm	6441	–
with ≥1 further CL measurement at 16–24 weeks	254	3.9% of the band
minimum CL <30 mm among those re-measured	47	18.5% (13.9–23.8)
minimum CL <25 mm among those re-measured	10	3.9% (1.9–7.1)
Birth <34 weeks, CL 25–29.9 mm	14/453	3.1% (1.7–5.1)
Birth <34 weeks, CL 30–34.9 mm	30/1363	2.2% (1.5–3.1)
Birth <34 weeks, CL ≥35 mm	41/2176	1.9% (1.4–2.5)

Repeat examinations were not scheduled systematically by the centre; most followed referral by the attending obstetrician or patient-initiated reassessment, often because of concern about cervical shortening (the reason for re-attendance is not recorded in the database). The subgroup is therefore clinically selected, and subsequent shortening cannot be distinguished from a short cervix missed at the index examination; the proportion of 3.9% must not be extrapolated to the whole band and does not estimate the false-negative rate of the transabdominal step. Although the preterm-birth rates at 30–34.9 mm and ≥35 mm do not suggest a marked excess in the 30–34.9 mm band, they cannot establish the number of short cervices missed by the initial protocol; published transabdominal–transvaginal differences are of the order of 2–5 mm (refs 25–28 of the main text). On the basis of these considerations the centre has adopted the 35 mm transabdominal trigger.
